# Supplementary material for: Transcriptomic Insights into Mechanisms of Early Seed Maturation in the Garden Pea (Pisum sativum L.)
Source: Cells. 2020 Mar 23;9(3):779. doi: 10.3390/cells9030779 (PMC7140803; doi:10.3390/cells9030779)

Sprint−2(20) vs Zhewa

Global Summary

%DE = 0.85

genes with fdr < 0.2 = 519 (233 + / 286 −)

genes with fdr < 0.1 = 464 (203 + / 261 −)

genes with fdr < 0.05 = 370 (146 + / 224 −)

genes with fdr < 0.01 = 274 (92 + / 182 −)

genes in genesets = 0

<FC> = 0

<t−score> = 11151.94

<p−value> = 0

<fdr> = 0.15

Global Genelist

Rank	ID	log(FC)	p−value	fdr / Metagene	Description
1	TRINITY_DN1	15.24	0e+00	0.00000020 x 1	
2	TRINITY_DN2	5.84	5e−09	0.00000020 x 1	
3	TRINITY_DN3	−18.92	7e−09	0.0000006 x 20	
4	TRINITY_DN1	−0.05	1e−08	0.0000034 x 12	
5	TRINITY_DN4	0	6e−08	0.0000037 x 10	
6	TRINITY_DN4	0	9e−08	0.00000310 x 7	
7	TRINITY_DN1	0	2e−07	0.0000039 x 11	
8	TRINITY_DN4	0.87	2e−07	0.00000314 x 1	
9	comp39525_c	0.23	2e−07	0.0000036 x 8	
10	TRINITY_DN7	0	2e−07	0.0000059 x 11	
11	TRINITY_DN4	0	4e−07	0.0000059 x 11	
12	TRINITY_DN1	−0.01	5e−07	0.0000059 x 11	
13	TRINITY_DN1	0	5e−07	0.0000059 x 11	
14	TRINITY_DN5	−0.23	5e−07	0.0000059 x 20	
15	TRINITY_DN1	0	6e−07	0.0000059 x 10	
16	TRINITY_DN1	0	6e−07	0.0000059 x 11	
17	TRINITY_DN1	−0.01	7e−07	0.00000512 x 8	
18	TRINITY_DN1	0	7e−07	0.0000081 x 11	
19	TRINITY_DN1	−2.13	8e−07	0.0000081 x 20	
20	TRINITY_DN4	0	1e−06	0.0000081 x 11	

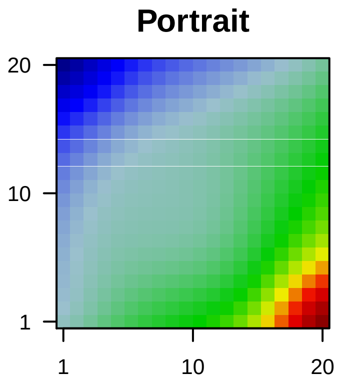

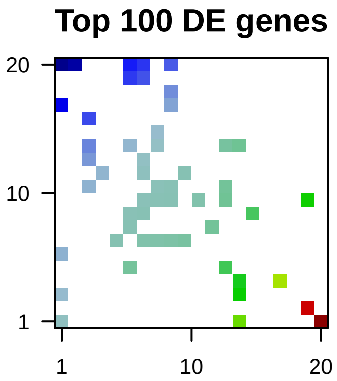

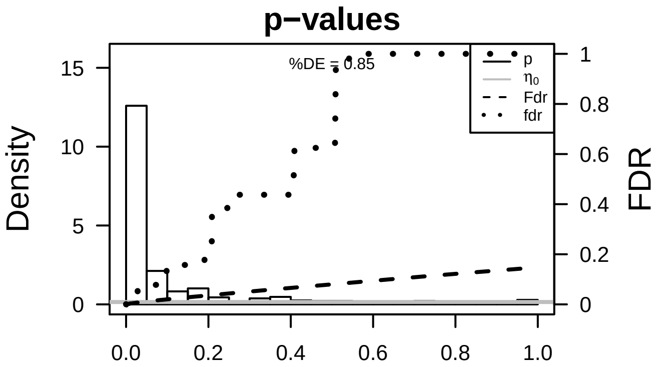

Supplement: Supplementary file 1 [file cells-09-00779-s001.zip › Supplementary materials/FileS3.zip!/FileS2/Hormones - Results/Summary Sheets - Differences/Reports/Sprint.2.20..vs.Zhewan.1.25..pdf]
